# *Mtrr* hypomorphic mutation alters liver morphology, metabolism and fuel storage in mice

**DOI:** 10.1016/j.ymgmr.2020.100580

**Published:** 2020-03-24

**Authors:** Alice P. Sowton, Nisha Padmanabhan, Simon J. Tunster, Ben D. McNally, Antonio Murgia, Aisha Yusuf, Julian L. Griffin, Andrew J. Murray, Erica D. Watson

**Affiliations:** a Department of Physiology, Development and Neuroscience, University of Cambridge, Cambridge, CB2 3EG, UK; b Department of Biochemistry, University of Cambridge, Cambridge, CB2 1GA, UK; c Centre for Trophoblast Research, University of Cambridge, Cambridge, CB2 3EG, UK; d Section of Biomolecular Medicine, Department of Metabolism, Digestion and Reproduction, Imperial College London, London, SW7 2AZ, UK

**Keywords:** One‑carbon metabolism, Liver metabolism, Hepatic fuel storage, Glycogen, Lipidomics, Mitochondrial function, 5-methyl-THF, 5-methyltetrahydofolate, *Agl*, amylo-alpha-1,6-glucosidase,4-alpha-glucanotransferase gene, BCA, bicinchoninic acid, *Bhmt*, betaine-homocysteine *S*-methyltransferase gene, CE, cholesteryl-ester, *Cebpa*, CCAAT/enhancer binding protein (C/EBP), alpha gene, Cer, ceramide, DAG, diacylglycerol, *Ddit3*, DNA damage inducible transcript 3 gene, ETS, electron transport system, FFA, free fatty acid, FCCP, *p*-trifluoromethoxyphenyl hydrazine, *G6pc*, glucose 6-phophastase gene, *Gbe1*, glycogen branching enzyme 1 gene, *Gsk3*, glycogen synthase kinase gene, *gt*, gene-trap, *Gyg*, glycogenin gene, *Gys2*, glycogen synthase 2 gene, HOAD, 3-hydoxyacyl-CoA dehydrogenase, *Isca1*, iron‑sulfur cluster assembly 1 gene, *J*O_2_, oxygen flux, LC-MS, liquid chromatography-mass spectrometry, LPC, lysophosphatidylcholine, *Mthfr*, methylenetetrahydrofolate reductase gene, *Mtr*, methionine synthase gene (also MS), *Mtrr*, methionine synthase reductase gene (also MSR), *Myc*, myelocytomatosis oncogene, NAFLD, non-alcoholic fatty liver disease, NASH, non-alcoholic steatohepatitis, *Ndufs*, NADH:ubiquinone oxidoreductase core subunit (ETS complex I) gene, OXPHOS, oxidative phosphorylation, PA, phosphatidic acid, PAS, periodic acid Schiff, PC, phosphatidylcholine, PE, phosphatidylethanolamine, PG, phosphatidylglycerol, PI, phosphatidylinositol, PIP, phosphatidylinositol phosphate(s), PS, phosphatidylserine, PL, phospholipid, RIPA, Radioimmunoprecipitation assay, SAH, S-adenosylhomocysteine, SAM, S-adenosylmethionine, SM, sphingomyelin, TAG, triacylglycerol, *Ugp2*, UDP-glucose pyrophophorylase 2 gene

## Abstract

Nonalcoholic fatty liver disease (NAFLD) is associated with dietary folate deficiency and mutations in genes required for one‑carbon metabolism. However, the mechanism through which this occurs is unclear. To improve our understanding of this link, we investigated liver morphology, metabolism and fuel storage in adult mice with a hypomorphic mutation in the gene methionine synthase reductase (*Mtrr*^*gt*^). MTRR enzyme is a key regulator of the methionine and folate cycles. The *Mtrr*^*gt*^ mutation in mice was previously shown to disrupt one‑carbon metabolism and cause a wide-spectrum of developmental phenotypes and late adult-onset macrocytic anaemia. Here, we showed that livers of *Mtrr*^*gt/gt*^ female mice were enlarged compared to control C57Bl/6J livers. Histological analysis of these livers revealed eosinophilic hepatocytes with decreased glycogen content, which was associated with down-regulation of genes involved in glycogen synthesis (e.g., *Ugp2* and *Gsk3a* genes). While female *Mtrr*^*gt/gt*^ livers showed evidence of reduced β-oxidation of fatty acids, there were no other associated changes in the lipidome in female or male *Mtrr*^*gt/gt*^ livers compared with controls. Defects in glycogen storage and lipid metabolism often associate with disruption of mitochondrial electron transfer system activity. However, defects in mitochondrial function were not detected in *Mtrr*^*gt/gt*^ livers as determined by high-resolution respirometry analysis. Overall, we demonstrated that adult *Mtrr*^*gt/gt*^ female mice showed abnormal liver morphology that differed from the NAFLD phenotype and that was accompanied by subtle changes in their hepatic metabolism and fuel storage.

## Highlights

• *Mtrr*^*gt*^ is a knockdown mutation in mice that disrupts one-carbon metabolism
• *Mtrr*^*gt/gt*^ female livers are enlarged, with eosinophilic hepatocytes
• Hepatic glycogen content and lipid metabolism is altered in *Mtrr*^*gt/gt*^ mice
• Mitochondrial electron transfer system activity was unaffected in *Mtrr*^*gt/gt*^ livers
• *Mtrr*^*gt/gt*^ liver phenotype is weaker than other mutations in one-carbon metabolism

## 1 Introduction

A connection between one‑carbon metabolism and fatty liver disease (steatosis) is well established [1]. This is largely based on studies showing that humans and rodents fed diets deficient for one‑carbon methyl groups (e.g., choline, folate, methionine, betaine) and/or have specific mutations in genes involved in one‑carbon metabolism (e.g., *Mat1a*, *Pemt*, *Gnmt*, *Mthfr*) develop fatty livers [[2], [3], [4], [5], [6], [7]]. Furthermore, patients with liver disease often have abnormal expression of key genes involved in methionine metabolism and hyperhomocysteinemia [8]. However, the mechanism through which this occurs is not well understood.

One‑carbon metabolism is at a major junction of several metabolic processes [9]. For instance, it has been implicated in hepatic lipid [10,11] and glycogen metabolism [5,12]. In liver, methyl groups from SAM are required for the sequential methylation of phosphatidylethanolamine (PE) to form phosphatidylcholine (PC) [13,14], the most abundant membrane phospholipid [15,16]. Liver cells have a high demand for PC synthesis, such that it requires as much as 40% of S-adenosylmethionine (SAM) for this purpose [17]. Disruption of hepatic PC production leads to steatosis in mice through accumulation of triacylglycerols (TAGs) and impaired secretion of very low-density lipoprotein particles [18,19]. Apart from hepatic lipid accumulation, glycogen storage disease is also associated with hyperhomocysteinemia [12] caused by perturbation of one‑carbon metabolism. Furthermore, mice that lack the enzyme glycine *N*-methyltransferase (GNMT), which regulates the ratio of SAM to S-adenosylhomocysteine (SAH) [20], develop glycogen storage disease in the liver [5]. Better understanding of how one‑carbon metabolism is connected to lipid and glycogen metabolism in the liver will help us gain insight into disease.

During one‑carbon metabolism, the enzyme methionine synthase (MTR, also known as MS), which contains a cobalamin (vitamin B_12_) cofactor, catalyzes the transfer of methyl groups from 5-methyltetrahydrofolate (5-methyl-THF) to cob(I)alamin to produce methylcobalamin. It donates this methyl group to methylate homocysteine to form methionine [21,22]. Methionine acts as a precursor for SAM, which then serves as a methyl donor for dozens of substrates including DNA, RNA, proteins (e.g., histones) and lipids (e.g., PE) [13,14,[23], [24], [25]]. In mammals, the cob(I)alamin cofactor of MTR becomes oxidized by molecular oxygen forming the inactive cob(II)alamin form. Methionine synthase reductase (MTRR or MSR) provides the electron that, along with the transfer of a methyl group from SAM, returns MTR to the active methylcobalamin form [26]. MTR is the only mammalian enzyme that uses 5-methyl-THF and deficiencies in MTR or MTRR causes an accumulation 5-methyl-THF that makes folate unavailable for other reactions, such as purine and pyrimindine biosynthesis [8,21]. Therefore, MTR and MTRR are key enzymes required for the progression of one‑carbon metabolism.

Mutations in human *MTR* and *MTRR* genes have not previously been associated with hepatosteatosis or glycogen storage disease. Mouse knockout mutations in *Mtr* and *Mtrr*, and other genes important for folate uptake and transport, are not conducive with life [21,[27], [28], [29]], resulting in embryonic lethality that prevents the assessment of liver disease in adulthood. We have developed a mouse model with a hypomorphic mutation in the *Mtrr* gene (*Mtrr*^*gt*^) that displays many key features of dietary folate deficiency or *MTRR* mutations in humans [[30], [31], [32], [33], [34]]. These include increased plasma total homocysteine concentrations [21,35], decreased plasma methionine concentrations [21], an altered hepatic SAM:SAH ratio [21], and tissue-specific dysregulation of DNA methylation [35]. These phenotypes are attributed to a substantial decrease in MTR activity [21]. We previously showed that *Mtrr*^*gt/gt*^ mice exhibit a wide spectrum and frequency of phenotypes during fetoplacental development (e.g. neural tube and heart defects, growth phenotypes), a portion of which are associated with embryonic lethality at midgestation [35]. The *Mtrr*^*gt/gt*^ mice that survive into adulthood display late onset macrocytic anaemia [36]. However, it is unknown whether adult livers of *Mtrr*^*gt/gt*^ mice display liver disease similar to other mouse models of defective one‑carbon metabolism. Here, we explored the effects of *Mtrr*^*gt/gt*^ homozygosity on glycogen and lipid storage and metabolism in adult livers. In doing so, we also investigated whether there were associated alterations in mitochondrial function that often accompany NAFLD and glycogen storage disease [[37], [38], [39], [40]].

## 2 Materials and methods

### 2.1 Mice and genotyping

All animal work was carried out in accordance with UK Home Office regulations under the Animals (Scientific Procedures) Act 1986 and underwent review by the University of Cambridge Animal Welfare and Ethical Review Body. The *Mtrr*^*gt*^ mouse line was generated through a gene-trap (gt) insertion into the *Mtrr* locus and upon germline transmission, the *Mtrr*^*gt*^ mutation was backcrossed to the C57Bl/6J genetic background for eight generations as previously described in detail [21,35]. C57Bl/6J mice (The Jackson Laboratory, Bar Harbour, ME, USA), which are wildtype for *Mtrr*, were used as controls for all experiments, and were maintained in house and bred separately from the *Mtrr*^*gt*^ mouse line. *Mtrr*^*gt/gt*^ mice were generated via *Mtrr*^*gt/gt*^ intercrosses. From birth, mice were housed in conventional cages in a temperature and humidity-controlled environment with a 12 h light/dark cycle. Mice were fed normal breeding diet (Rodent No. 3 breeding chow, Special Diet Services, Essex, UK) *ad libtum* from weaning onwards. A full dietary breakdown of the chow was previously described [35] including folic acid at 2.99 mg/kg of diet, methionine at 0.37%, choline at 1422.4 mg/kg of diet, and vitamin B_12_ at 19.2 μg/kg of diet. All female mice were virgins. DNA samples were obtained from ear tissue for PCR genotyping of the *Mtrr*^*gt*^ allele as reported earlier [35].

### 2.2 Tissue collection

Mice were euthanized by cervical dislocation at 9–16 weeks of age. Prior to dissection, mouse body weight was recorded. Whole livers were removed and weighed. The left lobe of each liver was dissected immediately in cold 1× phosphate buffered saline (1× PBS) diluted in diethyl-pyrocarbonate treated water and placed into ice-cold biopsy preservation medium (2.77 mM CaK_2_EGTA, 7.23 mM K_2_EGTA, 6.56 mM MgCl_2_.6H_2_O, 20 mM taurine, 15 mM phosphocreatine, 20 mM imidazole, 0.5 mM dithiothreitol, 50 mM MES, 5.77 mM Na_2_ATP, pH 7.1) for respirometry analysis. The right lateral lobe was prepared for histological analysis. The remaining liver tissue was snap-frozen in liquid nitrogen and stored at −80 °C for molecular analysis.

### 2.3 RNA extraction and quantitive reverse transcription PCR (RT-qPCR)

For analysis of folate metabolism and mitochondrial genes, tissue was homogenized using Lysing Matrix D beads (MP Biomedicals, Eschwege, Germany). Total RNA extraction from livers was completed using Trizol (Sigma-Aldrich) according to the manufacturer's instructions. All extracts were treated with DNase I (Thermo Fisher Scientific, Hemel Hempstead, UK). Reverse-transcription was performed with a RevertAid H Minus First Strand cDNA Synthesis Kit (Thermo Scientific) using 2 μg RNA in a 20-μl reaction. PCR amplification was conducted using MESA SYBR® Green qPCR MasterMix Plus (Eurogentec, Liege, Belgium) on a DNA Engine Opticon® 2 system (BioRad, Hercules, CA, USA). Transcript levels were normalized to *Hprt* or *Bactin* RNA levels. Fold change was quantified using the standard curve method. cDNA levels in C57Bl/6 J tissue were normalized to 1. Experiments were conducted in triplicate with at least three biological replicates.

For analysis of glycogen metabolism genes, RNA was extracted using the GenElute Mammalian Total RNA Miniprep Kit (Sigma-Aldrich) including OnColumn DNase treatment (Sigma-Aldrich). Reverse transcription was performed using SuperScript IV Reverse Transcriptase (Thermo Scientific) and Random Hexamers (Thermo Scientific) according to manufacturer's instructions using 1 μg total RNA as template. PCR amplification was performed using 2 μl of cDNA (diluted 1:10 in water) in a 10-μl reaction containing 1× DreamTaq Buffer (Thermo Scientific), 0.2 mM of each dNTP (Thermo Scientific), 0.5 μM of each primer (Sigma-Aldrich), 0.5 U DreamTaq (Thermo Scientific) and 0.1× SYBR® Green Dye (Thermo Scientific). Reactions were run in triplicate on a DNA Engine Opticon® 2 system (BioRad). Transcript levels were normalized to the geometric mean of *Hprt* and *Bactin* levels and fold change determined using the 2^-∆∆CT^ method with expression levels in C57Bl/6 J tissue normalized to 1.

Refer to Supplementary table 1 for primer sequences.

### 2.4 Tissue preparation and histological stains

Livers were fixed in 4% paraformaldehyde (PFA) in 1× PBS, embedded in paraffin using standard techniques and sectioned to 7 μm using a Leica RM2235 microtome (Leica Biosystems, Milton Keynes, UK). For each staining protocol, at least six histological sections from different regions of each liver (*N* = 3 female mice per genotype) were analysed. Tissue sections were stained with hematoxylin and eosin (H&E) or using a periodic acid-Schiff stain kit (Sigma-Aldrich, Gillingham, UK) according to standard protocols or the manufacturer's instructions. For immunohistochemistry, tissue sections were incubated in 3% H_2_O_2_ in 1× PBS for 30 min, treated with porcine trypsin tablets (Sigma-Aldrich) for 10 min, then incubated with blocking serum (5% donkey serum, 1% bovine serum albumin in 1× PBS) for 1 h. Tissue was then incubated in rabbit anti-mouse Ki67 IgG (abcam, Cambridge, UK, cat. No. ab15580, RRID:AB_443209) diluted to 1:100 in blocking serum overnight at 4 °C, then in donkey anti-rabbit IgG conjugated to horseradish peroxidase (abcam cat No. ab6802, RRID:AB_955445) diluted to 1:300 in blocking serum for 1 h at room temperature. Peroxidase substrate reactions were conducted with 3,3′-diaminobenzidine (DAB) chromagen substrate kit (abcam) according to the manufacturer's instructions. Sections were counterstained in Nuclear Fast Red (Sigma-Aldrich) before dehydration, clearing and coverslip mounting in DPX mountant (Sigma-Aldrich).

### 2.5 High-resolution respirometry analysis of mitochondrial function

From each left lobe of liver, 15–20 mg sections were cut, blotted dry and quickly weighed before immediate homogenisation in ice-cold respiratory medium (MiR05; 0.5 mM EGTA, 3 mM MgCl_2_.6H_2_O, 60 mM K-lactobionate, 20 mM taurine, 10 mM KH_2_PO_4_, 20 mM HEPES, 110 mM sucrose, 1 mg.ml^−1^ defatted bovine serum albumin, pH 7.1) at a final concentration of 100 μg.μl^−1^. Homogenisation was carried out using an Eppendorf pestle and a DLH overhead stirrer (Velp Scientifica, Usmate, Italy) with a maximum of five pulses of no longer than 5 s at 600 rpm until a consistent homogenate was generated. Liver homogenate (20 μl, equivalent to 2 mg wet tissue) was added to each chamber of an Oxygraph-2 k (Oroboros Instruments, Innsbruck, Austria) containing 2.1 ml MiR05 at 37 °C with continuous stirring. Oxygen flux (*J*O_2_) was continuously recorded using DatLab software 6.1 (Oroboros Instruments). Calibration at air saturation was carried out every day prior to experimentation and all data was corrected for background instrumental oxygen flux in accordance with the manufacturer instructions.

Respiration rates were analysed using a substrate-uncoupler titration that was run in duplicate on each biological replicate. *J*O_2_ through the N-pathway via electron transfer system (ETS) complex I was assayed in the LEAK state through addition of 1 mM malate and 10 mM glutamate (GM_*L*_). Subsequently, N-pathway supported oxidative phosphorylation (OXPHOS state) was stimulated through addition of 10 mM ADP (GM_*P*_). Maximal OXPHOS capacity was then assessed via addition of 10 mM succinate (GMS_*P*_), which can supply electrons through the S-pathway via complex II. Finally, the ETS was uncoupled from F_1_F_O_-ATP synthase through addition of the protonophore carbonyl cyanide *p*-trifluoro-methoxyphenyl hydrazone (FCCP) in 0.5 μM increments until maximal ETS capacity was observed (GMS_*E*_). Respiration rates were normalized to wet tissue mass and also to maximal ETS capacity (GMS_*E*_).

### 2.6 Citrate synthase and 3-hydroxyacyl-CoA dehydrogenase (HOAD) enzyme activity assays

Activities of citrate synthase (EC 2.3.3.1) and HOAD (EC 1.1.1.35) were analysed spectrophotometrically at 37 °C (Evolution 220 UV–Visible spectrophotometer, Thermo Fisher Scientific). Liver homogenate was generated from a small section of frozen tissue in 300 μl homogenisation buffer (20 mM HEPES, 1 mM EDTA, 0.1% *v/v* Triton X-100, pH 7.2) using a liquid nitrogen-cooled Eppendorf pestle. The supernatant was extracted following centrifugation (two spins at 380 x*g* for 30 s) and protein density quantified in each sample using a Pierce BCA Protein Assay Kit (Thermo Fisher Scientific) as per the manufacturer instructions. For citrate synthase activity, a sample containing 10 μg of protein was measured as previously described [41]. The citrate synthase assay buffer contained 18 mM tris-base, 0.1 mM 5,5′-dithiobis(2-nitrobenzoic acid) and 0.3 mM acetyl-CoA, pH 8.0 and the reaction stimulated by addition of 0.5 mM oxaloacetate. The change in absorbance was measured at 412 nm to determine citrate synthase activity. For HOAD activity, a sample containing of 20 μg protein was measured as previously described [42]. The HOAD assay buffer contained 50 mM imidazole, 0.1% *v/v* Triton X-100 and 0.15 mM NADH at pH 7.4. The reaction was initiated by addition of 0.1 mM acetoacetyl-CoA and absorbance change at 340 nm was measured. HOAD activity was corrected to citrate synthase activity to provide an indication of HOAD activity per mitochondrial unit.

### 2.7 Lipidomic and acyl-carnitine analysis by liquid chromatography-mass spectrometry (LC-MS)

Lipid metabolites were extracted from ~50 mg of frozen liver tissue using a modified Bligh and Dyer method [43]. Each sample was homogenized in 600 μl of 2:1 methanol/chloroform for 6 min at 20 Hz, and sonicated for 15 min. To each sample, 400 μl of water/chloroform (1:1) was added and samples were thoroughly vortexed before centrifugation (13,000 x*g*, 10 min). The aqueous and organic fractions were separated. To maximize metabolite yield, the extraction was repeated on the resultant protein pellet and the aqueous and organic fractions were separately pooled. Half of the resultant aqueous and organic fractions were combined for acyl-carnitine analysis and dried under nitrogen. To the remaining organic fraction, 250 μl of an internal standard mix containing 16 deuterated lipid standards was added before drying under nitrogen (see Supplementary table 2) for lipidomics.

For lipidomics, organic metabolites were reconstituted in 100 μl of methanol/chloroform (1:1) and 10 μl of this solution was diluted to 1:20 in propan-2-ol/acetonitrile/water (2:1:1). Open-profiling of lipid species was carried out using an Ultimate 3000 UHPLC system (Thermo Fisher Scientific) coupled to an LTQ Orbitrap Elite Mass Spectrometer (Thermo Fisher Scientific). Mass spectrometry was performed in both positive and negative modes. 5 μl of each sample was injected onto a C18 CSH column (2.1 × 100 mm, 1.7 μM pore size; Waters Ltd., Elstree, UK), which was held at 55 °C. The flow rate of the mobile phase gradient was 0.4 μl.min^−1^ with mobile phase A acetonitrile/water solution (3:2) and mobile phase B acetonitrile/propan-2-ol solution (1:9). The total run time was 20 min during which the mobile phases ran through the column in a gradient as follows: 40% B raised to 43% B at 2 min, 50% B at 2.1 min, 54% B at 12 min, 70% B at 12.1 min, 99% B at 18 min, and then 40% B for 2 min.10 mM ammonium formate (Fisher Scientific) was added to mobile phases A and B for positive mode, and 10 mM ammonium acetate (Sigma Aldrich) added for negative mode. Metabolites were ionised by heated electrospray for entry into the mass spectrometer with a source temperature of 420 °C and capillary temperature of 380 °C. Ionization voltages were set to 3.5 kV in positive mode and 2.5 kV in negative mode. Data was collected using the Fourier transform mass spectrometer analyser and the full scan was performed across an *m/z* range of 110–2000. Peaks were annotated by accurate mass using an automated in-house R script and comparison to the LipidMaps database [44]. Where isobaric species of different classes were annotated, lipid identity was determined through manual identification of fragmentation patterns according to published methods [[45], [46], [47]].

The combined carnitine extraction fraction was reconstituted in 150 μl of a methanol/water (4:1) solution containing an internal standard mix of eight deuterated acyl carnitines at a concentration of 2 μM (see Supplementary table 2). The metabolite levels were quantified using a Vanquish UHPLC^+^ series (Thermo Fisher Scientific) fitted with an ACE Excel-2 C18-PFP 5 μm column (100 Å, 150 × 2.1 mm; Advanced Chromatography Technologies Ltd.) conditioned at 40 °C, coupled to a TSQ Quantiva Triple Quadripole Mass Spectrometer (Thermo Fisher Scientific). The mobile phase was pumped at 0.45 ml.min^−1^ with mobile phase A 0.1% aqueous formic acid solution and mobile phase B 0.1% formic acid/methanol solution. Mobile phase B was initially held at 0.5% for 1 min and was then subjected to a linear increase to 100% B over 9 min, held for 2 min, and then brought back to initial conditions over 0.1 min. UPLC-MS data acquisition and processing was carried out using Xcalibur software (version 2.2, Thermo Fisher Scientific) with putative recognition of the detected metabolites performed using a targeted MS/MS analysis.

The protein pellet remaining from the metabolite extraction was homogenized in 250 μl of RIPA buffer and used to spectrophotometrically quantify the protein concentration using a Pierce BCA protein assay kit (Sigma-Aldrich) as per the manufacturer's instructions. All metabolite levels were then normalized to the intensity of an appropriate internal standard and to the protein concentration of the sample to account for differences in initial sample size.

### 2.8 Hepatic glycogen concentration

Glycogen extraction from livers was carried out as previously described [48]. Glycogen was extracted from ~100 mg of liver from each sample and resuspended in 1 ml of H_2_O. Glycogen content was measured with a spectrophotometer (490 nm absorbance; Elx800 microplate reader, BioTek, Winooski, VT, USA) using the phenol-sulphuric acid method [4]. A standard curve was plotted to calculate glycogen concentrations using serial dilution of rabbit liver glycogen (Sigma-Aldrich). Glycogen content was measured in duplicate with 6–8 biological replicates per genotype and sex.

### 2.9 Statistics, equipment and software

Statistical analyses were performed using GraphPad Prism 6 software (La Jolla, CA, USA). Differences attributable to genotype, sex, or the interaction of these two factors were assessed through a two-way analysis of variance (ANOVA) whilst differences between C57Bl/6 J and *Mtrr*^*gt/gt*^ mice within one sex were separately analysed either through a two-tailed, unpaired Mann-Whitney test or a two-tailed, unpaired student's *t*-test. *P* < .05 was considered significant. A NanoZoomer 2.0-RS (Hamamatsu) and NDP.view2 viewing software program (Hamamatsu) were used to obtain images. Histological measurements including Ki67^+^ and PAS^+^ cell counts and percentage area of PAS stain were performed using ImageJ software (NIH, Bethesda, MD, USA). All graphs were produced using GraphPad Prism 6 software. PCR primers were designed using Primer-BLAST software [49].

## 3 Results

### 3.1 Misexpression of genes that encode for folate metabolic enzymes in *Mtrr*^*gt/gt*^ female liver

**Fig. 1:**
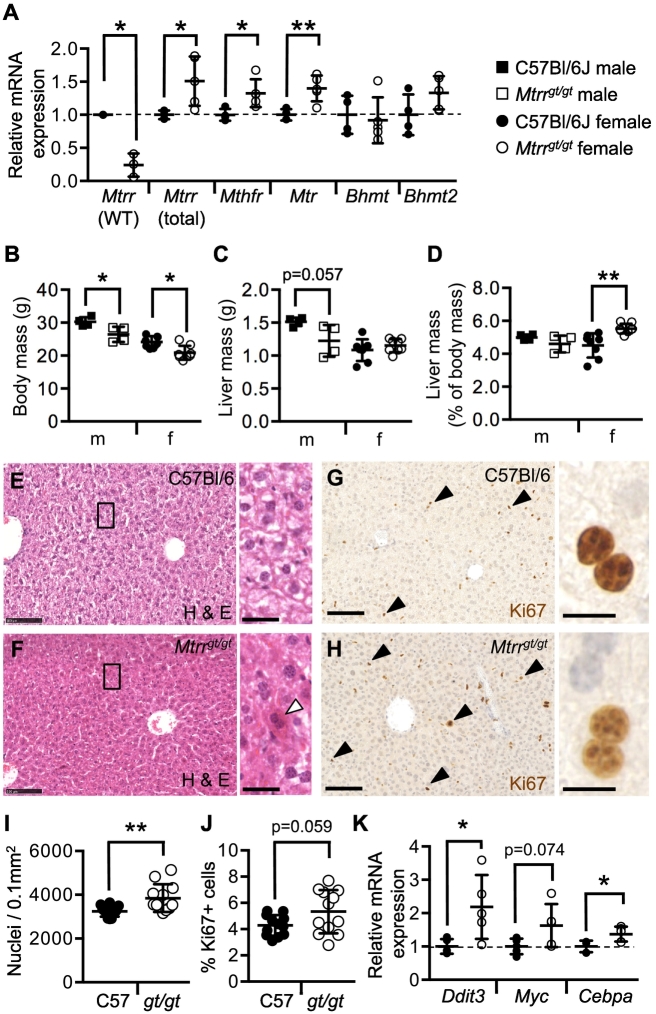
*Mtrr*^*gt/gt*^ female mouse livers are large with increased eosinophilia in hepatocytes. **(A)** Relative mRNA expression of genes encoding for folate metabolism enzymes in C57Bl/6J control and *Mtrr*^*gt/gt*^ livers of female mice as determined by RT-qPCR analysis. *N* = 3–5 females per genotype. *Mtrr* WT, wildtype *Mtrr* transcript; *Mtrr* total, wildtype *Mtrr* transcript plus gene-trapped *Mtrr* transcript. Data is presented as fold change relative to C57Bl/6J controls (normalized to 1; mean ± sd). Independent *t*-tests, *p < .05, **p < .01. (**B**) Body mass and **(C)** liver mass in C57Bl/6J control and *Mtrr*^*gt/gt*^ male (m) and female (f) mice. **(D)** Liver mass as a percentage of body mass in male (m) and female (f) C57Bl/6 J and *Mtrr*^*gt/gt*^ mice. *N* = 4–8 mice per sex per genotype. Histological sections of C57Bl/6J and *Mtrr*^*gt/gt*^ female livers stained with **(*E*-F)** hematoxylin (blue) and eosin (pink; H&E) or **(G-H)** an antibody against Ki67 protein (brown; nuclei counterstained with hematoxylin [blue]). White arrowhead indicates a strongly eosinophilic hepatocyte. Black arrowheads indicate Ki67^+^ cells. Black boxes indicate regions shown in higher magnification to right. Scale bars: low magnification, 100 μm; high magnification, (E-F) 25 μm and (G-H) 12.5 μm. **(I)** Nuclear density in histological sections of female C57Bl/6J and *Mtrr*^*gt/gt*^ livers as represented the number of cells per mm^2^. **(J)** Mitotic cell counts in histological sections of livers from C57Bl/6J and *Mtrr*^*gt/gt*^ female mice as determined by the percentage of hepatocytes that were Ki67^+^. At least four liver regions were assessed per female. *N* = 3 females per genotype. Independent *t*-test, ^§^*p* = .0592, **p < .01. (**K**) Relative mRNA expression in C57Bl/6J control and *Mtrr*^*gt/gt*^ female livers of *Ddit3*, *Myc*, and *Cebpa* genes as determined by RT-qPCR analysis. N = 3–5 females per genotype. Data is presented as fold change relative to C57Bl/6J controls (normalized to 1; mean ± sd). Independent *t*-tests, *p < .05. Legend: Squares, males; Circles, females; black, C57Bl/6J; white, *Mtrr*^*gt/gt*^.

The *Mtrr*^*gt*^ allele is a knock-down mutation that leads to the disruption of one‑carbon metabolism in liver including a 62% reduction in MTR activity associated with plasma hyperhomocysteinemia and tissue-specific alterations in DNA methylation [21,35]. Using RT-qPCR, we confirmed that the wildtype *Mtrr* transcript was significantly reduced in *Mtrr*^*gt/gt*^ female liver to 24.1 ± 10.0% (mean ± sd) of C57Bl/6J control transcript levels (*p* = .0016; Fig. 1A). This result was in line with a previous report, which also showed a corresponding reduction in wildtype MTRR protein expression in *Mtrr*^*gt/gt*^ livers [21]. Of note, *Mtrr*^*gt/gt*^ female livers displayed elevated expression of total *Mtrr* transcripts (including wildtype and gene-trapped *Mtrr* transcripts) relative to controls (150.4 ± 37.3% of C57Bl/6J transcripts, *p* = .033; Fig. 1A), which was different to a previous study [21] and suggested a potential transcriptional regulatory feedback loop. Differences in total *Mtrr* RNA expression levels in *Mtrr*^*gt/gt*^ livers between studies might be due to the controls used: C57Bl/6J livers [this study] versus *Mtrr*^*+/+*^ littermates [21]. Similarly, other genes that encode for folate metabolism enzymes in *Mtrr*^*gt/gt*^ female livers were also up-regulated, such as *Mthfr* and *Mtr* (132.7 ± 21.0% [p = .033] and 139.9 ± 19.5% [*p* = .024] of C57Bl/6J transcripts, respectively; Fig. 1A). However, the expression of *Bhmt* and *Bhmt2* genes, which provide an alternate pathway for methylation of homocysteine [50], was unchanged in *Mtrr*^*gt/gt*^ female livers compared to controls (Fig. 1A). Altogether, these data suggest possible transcriptional compensation for *Mtrr* knock-down via the selective up-regulation of other genes involved in one‑carbon metabolism. Whether these transcriptional changes lead to alterations in protein level or function requires confirmation.

### 3.2 Hepatocytes are eosinophilic in *Mtrr*^*gt/gt*^ female mice

To explore the effects of the *Mtrr*^*gt*^ mutation on body mass, male and female *Mtrr*^*gt/gt*^ mice were weighed and compared to C57Bl/6J controls (*N* = 4–8 mice per group). *Mtrr*^*gt/gt*^ homozygosity resulted in low body weight in male and female mice relative to controls (*p* < .05, Fig. 1B). Next, liver mass was assessed. While *Mtrr*^*gt/gt*^ males showed a proportional decrease in liver and body mass (*p* = .343), *Mtrr*^*gt/gt*^ female livers represented a greater percentage of body mass than controls (*p* = .0006; Fig. 1C-D). This result indicated that the *Mtrr*^*gt/gt*^ mutation led to a female-specific effect on liver size. Histological analysis of female livers showed that *Mtrr*^*gt/gt*^ hepatocytes were more densely packed than controls (Fig. 1E-F) with greater nuclear density (nuclei per 0.1 mm^2^; p < .05) (Fig. 1I). It is possible that this was a result of increased cell proliferation since the percentage of mitotic Ki67^+^ cells was slightly higher in *Mtrr*^*gt/gt*^ female livers (*p* = .059; Fig. 1G-H,J). However, the expression of genes that regulate cell proliferation in the liver (e.g., *Myc* [*p* = .0746] and *C/ebpa* [*p* = .0184]; Fig. 1K) was not appreciably higher (1.3–1.6-fold change) in *Mtrr*^*gt/gt*^ female livers relative to controls. Besides being more densely arranged, *Mtrr*^*gt/gt*^ hepatocytes were eosinophilic (Fig. 1E-F), which might indicate an aberrant accumulation of proteins [51] caused by cell stress [52] or altered glycogen or lipid content (see Sections 3.3 and 3.4). We observed that the cell stress gene *Ddit3* was up-regulated by 2.2-fold in *Mtrr*^*gt/gt*^ female livers compared to controls (*p* = .0271; Fig. 1K). Therefore, the *Mtrr*^*gt/gt*^ mutation might activate stress pathways in liver.

### 3.3 Analysis of glycogen storage in *Mtrr*^*gt/gt*^ livers

**Fig. 2:**
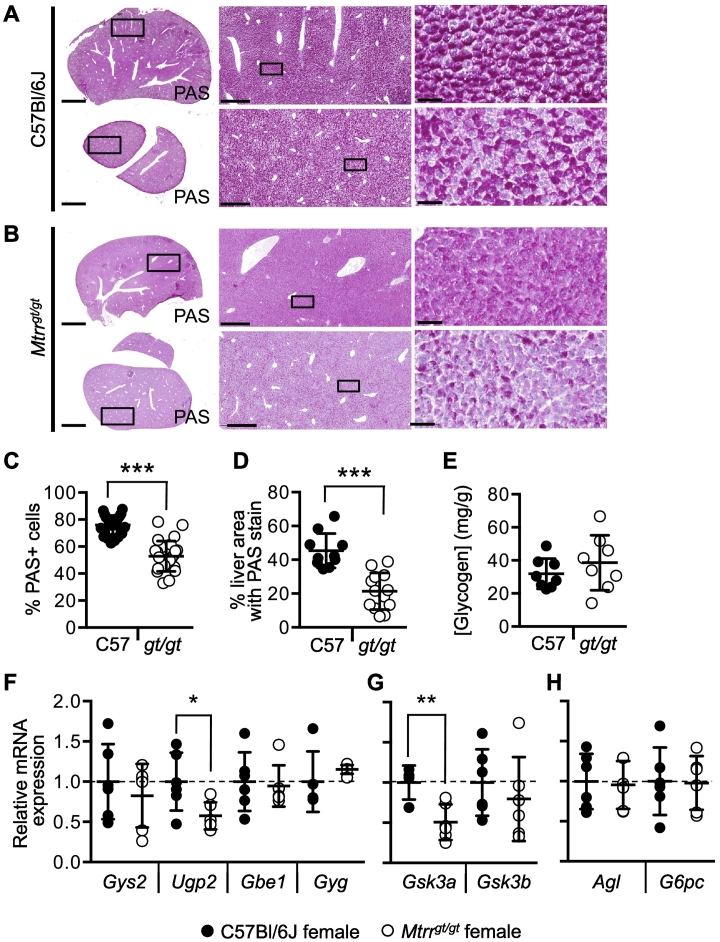
*Mtrr*^*gt/gt*^ livers from female mice show altered glycogen storage. Representative histological sections showing two liver regions of (**A**) C57Bl/6J and (**B**) *Mtrr*^*gt/gt*^ female mice stained with periodic acid-Schiff (PAS, dark pink; nuclei are counterstained with hematoxylin [blue]). Black boxes indicate regions shown in higher magnification to right. Scale bars: lowest magnification, 2.5 mm; middle magnification, 500 μm; high magnification, 50 μm. Graphs showing (**C**) the percentage of PAS^+^ liver cells and (**D**) the intensity of PAS staining (represented by the percentage of image area with PAS stain) in histological sections of C57Bl/6J livers and *Mtrr*^*gt/gt*^ livers of female mice. At least four liver regions were assessed per female. N = 3 females per genotype. Data is represented as mean ± sd. Independent t-test, ***p < .005. (**E**) Hepatic glycogen concentration in female C57Bl/6J and *Mtrr*^*gt/gt*^ mice. *N* = 8 livers per group. Data is represented as mean ± sd. Independent t-test. (**F—H**) Relative mRNA expression in C57Bl/6J control and *Mtrr*^*gt/gt*^ female livers was determined for (**F**) *Gys2*, *Ugp2*, *Gbe1* and *Gyg*, (**G)***Gsk3a* and *Gsk3b* and (**H**) *Agl* and *G6pc* as determined by RT-qPCR analysis. *N* = 6 livers per group. Data is presented as fold change relative to C57Bl/6J controls (normalized to 1; mean ± sd). Independent t-tests, ^§^*p* = .0599, *p < .05, **p < .01, ***p < .001. Black circles, C57Bl/6J; white circles, *Mtrr*^*gt/gt*^.

Next, hepatic fuel storage was investigated in *Mtrr*^*gt/gt*^ female livers. First, periodic acid-Schiff (PAS) staining was performed on histological sections of C57Bl/6J and *Mtrr*^*gt/gt*^ female livers to assess glycogen storage (*N* = 3 livers per genotype). Fewer *Mtrr*^*gt/gt*^ hepatocytes stained positive for PAS (52.8 ± 11.2%, *p* < .0001) compared with controls (76.0 ± 7.0%; Fig. 2A-C), and positive cells showed less intense PAS stain in *Mtrr*^*gt/gt*^ livers (Fig. 2A-B,D). However, when glycogen was extracted from C57Bl/6J and *Mtrr*^*gt/gt*^ female livers, direct biochemical quantification of liver glycogen revealed that there was no difference in hepatic glycogen concentration in *Mtrr*^*gt/gt*^ female livers compared to controls (Fig. 2E). These results indicated a discrepancy between the histology and biochemical assay that might be explained by a degree of inter-individual variability caused by ad libitum feeding or by the fact that both the PAS stain and the phenol-sulphuric acid method are not specific for glycogen [53]. However, the discrepancy observed is unlikely to be due to major differences in glycogen content between liver lobes [54].

To better understand the effects of the *Mtrr*^*gt*^ mutation on hepatic glycogen metabolism, expression of genes involved in glycogen synthesis (i.e., *Gys2*, *Ugp2*, *Gbe1*, *Gyg*, *Gsk3a*, and *Gsk3b*), and glycogen breakdown (i.e., *Agl* and *G6pc*) were assessed via RT-qPCR in C57Bl/6J and *Mtrr*^*gt/gt*^ female livers. Glycogen synthesis regulator genes *Ugp2* and *Gsk3a* were down-regulated by ~2-fold in *Mtrr*^*gt/gt*^ female livers compared to C57Bl/6J controls (*p* < .002; Fig. 2F, G). All other genes assessed displayed normal expression in *Mtrr*^*gt/gt*^ female livers compared to controls (Fig. 2F-H). UGP2 is an enzyme responsible for converting glucose-1-phosphate to UDP-glucose, the precursor of glycogen. In contrast, GSK3 negatively regulates glycogen synthesis [55,56] and reduced expression of its gene in *Mtrr*^*gt/gt*^ livers might indicate transcriptional feedback associated with reduced hepatic glycogen. Down-regulation of these genes in *Mtrr*^*gt/gt*^ female livers substantiates the histological data that showed reduced glycogen (Fig. 2B). Overall, these data revealed dysregulation of hepatic glycogen synthesis in *Mtrr*^*gt/gt*^ females. However, to more fully understand the effect of the *Mtrr*^*gt*^ allele on hepatic glycogen metabolism, protein levels should be assessed.

### 3.4 Fatty acid metabolism but not lipid storage was altered in *Mtrr*^*gt/gt*^ livers

**Fig. 3:**
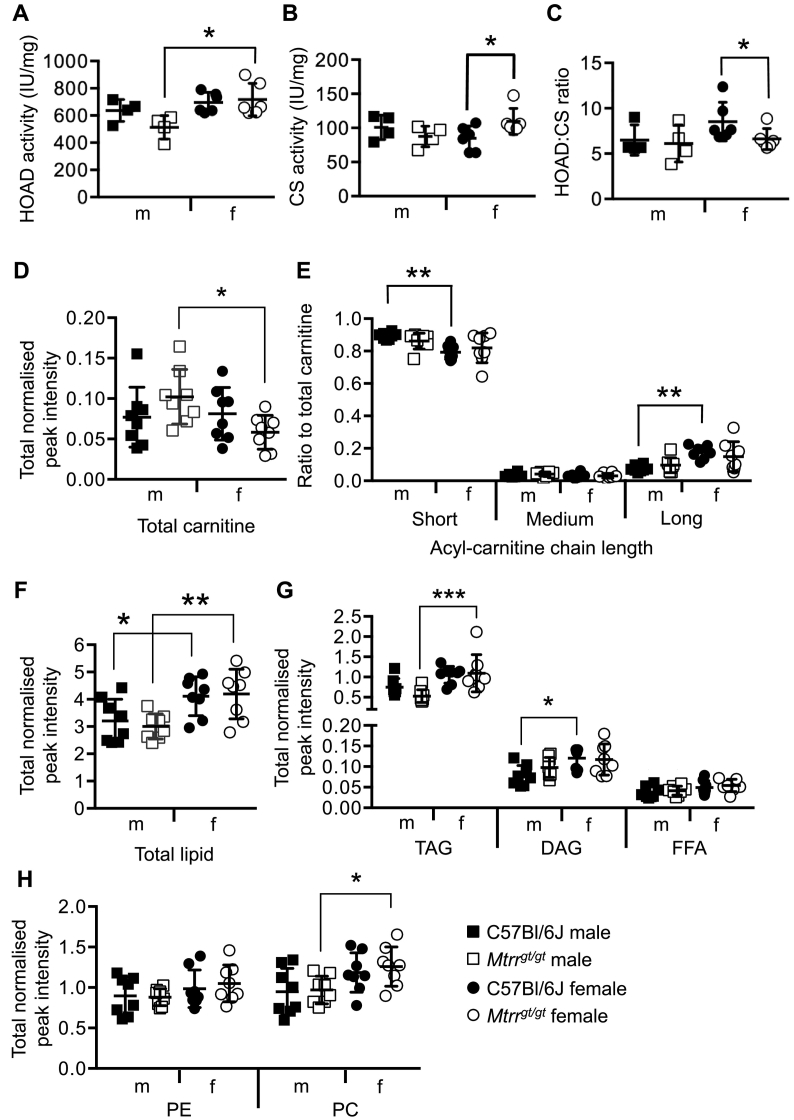
*Mtrr*^*gt/gt*^ female mouse livers show altered fatty acid metabolism and not storage of lipids. (**A**) 3-hydroxyacyl-CoA dehydrogenase (HOAD) enzyme activity, (**B**) citrate synthase (CS) activity, and (**C**) HOAD activity relative to CS activity in male (m) and female (f) C57Bl/6J and *Mtrr*^*gt/gt*^ mouse liver. *N* = 4–6 livers per group, mean ± sd. Two-way ANOVA, Sidak's multiple comparison, *p < .05. (**D**) Total carnitine species and (**E**) relative levels of short-chain (C2-C5), medium-chain (C6-C12) and long-chain (≥C13) acyl-carnitines to the total carnitine pool as measured by liquid chromatography-mass spectrometry (LC-MS) in male (m) and female (f) C57Bl/6J and *Mtrr*^*gt/gt*^ mouse liver. (**F**) Total detected lipid, (**G**) levels of triacylglycerol (TAG), diacylglycerol (DAG) and free fatty acids (FFAs) and (**H**) levels of phosphatidylethanolamine (PE) and phosphatidylcholine (PC) measured through open profiling lipidomics by LC-MS in male (m) and female (f) C57Bl/6J and *Mtrr*^*gt/gt*^ mouse liver. See also Supplementary fig. 1. Data in panels **D-H** are normalized to an appropriate internal standard and sample protein concentration. N = 8 livers per group, mean ± sd. *p < .05, **p < .01, ***p < .001, Two-way ANOVA, Sidak's multiple comparison. Squares, males; Circles, females; black, C57Bl/6J; white, *Mtrr*^*gt/gt*^.

To further explore the effects of the *Mtrr*^*gt*^ allele on hepatic fuel storage, lipid metabolism was investigated. Firstly, 3-hydoxyacyl-CoA dehydrogenase (HOAD) activity was measured spectrophotometrically in male and female liver to indicate whether β-oxidation of fatty acids was altered by the *Mtrr*^*gt*^ mutation. Regardless of sex, HOAD activity was similar between C57Bl/6J control and *Mtrr*^*gt/gt*^ livers (Fig. 3A). Interestingly, HOAD activity in *Mtrr*^*gt/gt*^ female livers was 139.9% ± 33.3% (mean ± sd) of the activity in *Mtrr*^*gt/gt*^ males (*p* = .008; Fig. 3A), yet this sexually dimorphic effect was not observed in controls (Fig. 3A). When HOAD activity was normalized to citrate synthase activity, a putative marker of mitochondrial content [57], *Mtrr*^*gt/gt*^ female livers displayed 22.5% less activity than C57Bl/6J female livers (*p* = .026; Fig. 3B-C). However, no differences were apparent in males (**Fig. 3B—C**). Overall, these data suggested that the *Mtrr*^*gt/gt*^ mutation in mice might cause altered hepatic fatty acid metabolism in a sexually dimorphic manner.

To determine whether reduced fatty acid metabolism in *Mtrr*^*gt/gt*^ female livers resulted in lipid accumulation, liver lipidomic analysis was performed in C57Bl/6J and *Mtrr*^*gt/gt*^ mice by LC-MS. Firstly, we observed that the level of total carnitines (including free carnitine) and the relative proportions of short- (C2–5), medium- (C6–12) and long-chain (≥C13) acyl-carnitines of male and female *Mtrr*^*gt/gt*^ livers were comparable to the same-sex control group (Fig. 3D-E). While further considering sex-specific differences, we observed that short-chain acyl-carnitine levels were lower and long-chain acyl-carnitine levels were higher in control female livers than male livers (*p* < .05; two-way ANOVA, Sidak's multiple comparison; Fig. 3E). This was not apparent in *Mtrr*^*gt/gt*^ mice (Fig. 3D), which only displayed sexual dimorphism of hepatic total carnitine, an effect that was absent in controls: *Mtrr*^*gt/gt*^ female livers had 43.1% less total carnitine (*p* = .045) than *Mtrr*^*gt/gt*^ male livers (two-way ANOVA, Tukey's multiple comparison; Fig. 3E). Taken together with the HOAD activity data (Fig. 3C), these data supported the hypothesis that fatty acid handling was differently affected in *Mtrr*^*gt/gt*^ mice in a sexually dimorphic manner.

Next, open profiling of the hepatic lipidome was performed. Total lipid content was unchanged in *Mtrr*^*gt/gt*^ livers compared to same-sex C57Bl/6J controls (Fig. 3F). Yet, there was a distinct sex-specific effect of total lipid content regardless of genotype (Fig. 3F) that is consistent with other studies [58,59]. For instance, C57Bl/6J female livers showed 22.0% more total lipid than C57Bl/6J males (*p* = .041) and similarly, *Mtrr*^*gt/gt*^ females livers had 28.3% more total lipid content than *Mtrr*^*gt/gt*^ males (*p* = .007; two-way ANOVA, Sidak's multiple comparison). Lipid species were grouped by lipid class for further analysis. Similar to total lipid content, levels of TAGs, DAGs, FFAs, cholesteryl-esters, ceramides, sphingomyelins, and phospholipids were unchanged in male and female *Mtrr*^*gt/gt*^ livers when compared to same sex controls (Fig. 3G-H, Supplementary fig. 1A). However, there was a sex-specific difference between TAGs levels regardless of genotype whereby female livers consistently showed higher levels of specific medium-chain (48–54 carbon) and long-chain (>54 carbon) TAGs (*p* < .05; two-way ANOVA with a Holm-Sidak correction for false discoveries, Sidak's multiple comparison; Supplementary fig. 2). The medium-chain TAGs were species rich in palmitic and oleic acid (Supplementary fig. 2A) and are associated with de novo lipogenesis [60]. This data might account for the increased total lipid content that was observed in the female livers.

In specific cases, such as in DAGs and phosphatidylinositol phosphates, there was a sex-specific effect in C57Bl/6 J livers (p < .05, two-way ANOVA, Sidak's multiple comparison) that was absent in *Mtrr*^*gt/gt*^ livers (Fig. 3G, Supplementary fig. 1B-C). Conversely, there were several lipid species whereby a sex-specific effect in liver was generated by the *Mtrr*^*gt/gt*^ mutation that was not apparent in controls. More specifically, *Mtrr*^*gt/gt*^ female livers contained more TAG, phosphatidylcholine and phosphatidic acid than male *Mtrr*^*gt/gt*^ livers (p < .05, two-way ANOVA, Sidak's multiple comparison; Fig. 3G-H**,**Supplementary fig. 1B). Sexual dimorphism of the hepatic lipidome suggested that male and female mice might be differently affected by the *Mtrr*^*gt*^ mutation. Overall, these data suggested that *Mtrr*^*gt/gt*^ homozygosity in female liver might alter fatty acid metabolism without significantly affecting in lipid content.

### 3.5 *Mtrr*^*gt/gt*^ livers display normal mitochondrial electron transfer system function

**Fig. 4:**
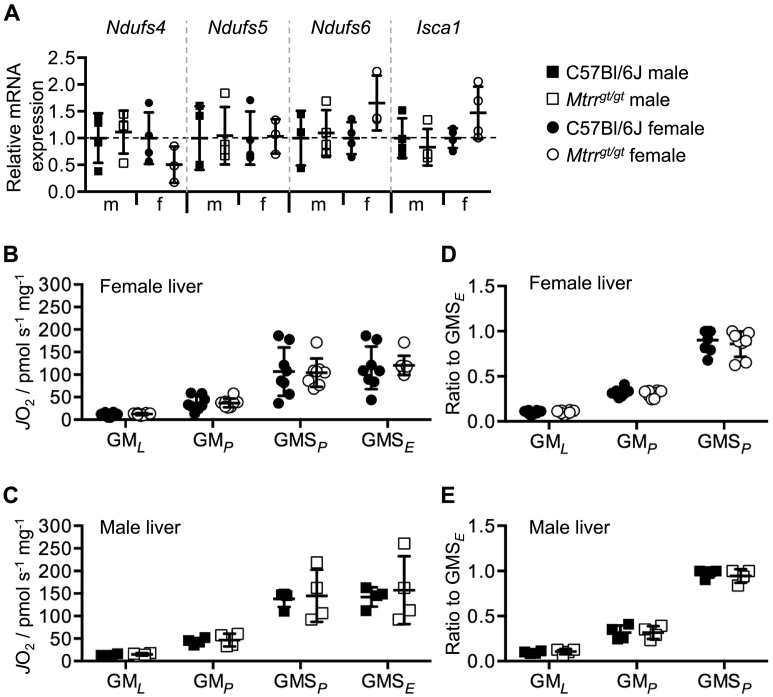
Mitochondrial function is unchanged in livers of *Mtrr*^*gt/gt*^ adult mice. (**A**) Relative mRNA expression of genes encoding for nuclear-encoded mitochondrial genes in male (m) and female (f) livers from C57Bl/6J control and *Mtrr*^*gt/gt*^ adult mice as determined by RT-qPCR analysis. *N* = 3–5 females per genotype. Data is presented as fold change relative to C57Bl/6J controls (normalized to 1; mean ± sd). (**B-E**) Mitochondrial respiratory function (*J*O_2_) in male and female livers from C57Bl/6J and *Mtrr*^*gt/gt*^ adult mice. GM_*L*_, malate and glutamate stimulate LEAK respiration through the N-pathway via complex I; GM_*P*_, OXPHOS supported by glutamate and malate through the N-pathway via complex I; GMS_*P*_, OXPHOS supported by glutamate, malate and succinate through the NS-pathway via complexes I and II; GMS_*E*_, maximal ET capacity supported by malate, glutamate and succinate through the NS-pathway via complexes I and II in uncoupled mitochondria. Oxygen flux rates were normalized to (**B-C**) wet tissue mass or to (**D-E**) GMS_*E*_ to indicate mitochondrial respiratory capacity per mitochondrial unit. Squares, males; circles, females; black, C57Bl/6J; white, *Mtrr*^*gt/gt*^.

Lastly, we aimed to assess whether the metabolic phenotype of *Mtrr*^*gt/gt*^ livers was related to changes in mitochondrial respiratory function. First, the mRNA expression of a selection of nuclear-encoded genes required for mitochondrial function (e.g., *Ndufs4*, *Ndufs5*, *Ndufs6*, *Isca1*) was assessed. No significant differences in mRNA expression between male and female C57Bl/6 J and *Mtrr*^*gt/gt*^ livers were observed (*p* > .114; Fig. 4A). Next, mitochondrial respiration rates were analysed in fresh C57Bl/6 J and *Mtrr*^*gt/gt*^ liver homogenates by high-resolution respirometry (*N* = 4 males and *N* = 8 females per genotype). No difference in flux was observed in livers of male or female *Mtrr*^*gt/gt*^ mice compared with controls, regardless of the state assayed (when corrected to wet tissue mass; Fig. 4B-C). Similarly, when rates were corrected to GMS_*E*_ (maximal ETS capacity) to provide a qualitative indication of changes in mitochondrial function per mitochondrial unit, oxygen flux rates were no different to controls **(**Fig. 4D-E). Altogether, these data suggest that the *Mtrr*^*gt/gt*^ mutation does not cause mitochondrial dysfunction in the liver of adult mice.

## 4 Discussion

The *Mtrr*^*gt*^ allele in mice causes defects in one‑carbon metabolism including plasma hyperhomocysteimia, decreased plasma methionine, increased tissue 5-methyl-THF, and altered cellular methylation [21,35]. Similar to folate deficiency and *MTRR* mutations in humans [30,[32], [33], [34]], metabolic derangement caused by *Mtrr*^*gt/gt*^ homozygosity in mice is associated with macrocytic anaemia [36] and a wide spectrum of developmental phenotypes (e.g., neural tube defects, cardiac defects, fetal growth restriction) that display incomplete penetrance [35]. Here, we investigated the effects of the *Mtrr*^*gt*^ allele on liver metabolism given that NAFLD has previously been associated with defects in one‑carbon metabolism [[2], [3], [4], [5], [6], [7], [8]] and to better understand the larger metabolic milieu in *Mtrr*^*gt/gt*^ mice. We found adult *Mtrr*^*gt/gt*^ female livers were large and displayed eosinophilic hepatocytes, the appearance of which was distinct from the lipid-filled vacuoles of hepatocytes associated with steatosis caused by dietary folate deficiency and/or *Mthfr* knockout mutation in mice [31,61]. Despite this, the *Mtrr*^*gt*^ mutation leads to changes in hepatic glycogen storage and β-oxidation of fatty acids that occur independent of changes in electron transfer system capacity.

The most striking phenotype of this study was the densely packed, eosinophilic hepatocytes in *Mtrr*^*gt/gt*^ female mice. The altered appearance of *Mtrr*^*gt/gt*^ hepatocytes might be explained by impaired glycogen storage. Evidence for this hypothesis was reduced glycogen content in *Mtrr*^*gt/gt*^ female livers as determined by PAS stain in combination with a down-regulation of genes involved in glycogen synthesis. Indeed, mice fed a folate- and/or choline-deficient diet exhibit alterations in hepatic DNA methylation of genomic regions that are associated with genes related to carbohydrate metabolism [62]. In another context, folate transporters (e.g., FOLR1) and metabolic enzymes (i.e., MTR and MTHFR) are highly expressed in specialized placental cells involved in glycogen storage and metabolism [63]. These observations support the hypothesis that glycogen metabolism and folate metabolism might be linked.

In conflict with the data above, glycogen concentration was unchanged in *Mtrr*^*gt/gt*^ livers compared to controls when biochemically determined. While different liver lobes were assessed in the PAS stain and the phenol-sulphuric acid method, glycogen content is typically consistent between lobes [54] and is unlikely the cause of this discrepancy. Given that glycogen is a multi-branched polysaccharide of glucose, it is possible that the structure of glycogen is altered (e.g., branch lengths) in *Mtrr*^*gt/gt*^ livers. As a result, PAS stain and phenol-sulphuric acid method might differently recognize the glycogen pool based on these hypothetical structural changes. Alternatively, PAS stain also detects glycoproteins and proteoglycans in addition to glycogen [64]. Therefore, changes in PAS staining intensity in *Mtrr*^*gt/gt*^ female livers might instead reflect major cellular changes, such as in connective tissue and basal laminae. More sensitive assays, such as ^13^C nuclear magnetic resonance [65] or amyloglucosidase digestion followed by liquid chromatography/electrospray ionization–tandem mass spectrometry quantification of the resultant free glucose [66] might reveal more accurate changes in *Mtrr*^*gt/gt*^ liver glycogen content. Additionally, *Mtrr*^*gt/gt*^ mice that are challenged by an obesogenic diet might show more striking and consistent defects in glycogen synthesis and/or metabolism relative to controls. This type of increased sensitivity to dietary stress should be explored in the future.

Independent of glycogen storage, eosinophila of *Mtrr*^*gt/gt*^ hepatocytes might be explained by aggregation of non-specific cytoplasmic proteins [51]. Endoplasmic reticulum (ER) stress, a condition where unfolded and misfolded proteins accumulate, is a feature of fibroblasts of human patients with mutations in *MTR* [67]. These cells display mislocalization of RNA binding proteins that prevent nucleo-cytoplasmic shuttling of RNA and splicing of mRNA [67], which likely has a profound effect on post-transcriptional regulation and normal cell function. Hyperhomocysteinemia, which is a prominent feature in *Mtrr*^*gt/gt*^ mice [21,35], associates with ER stress in other contexts [68,69]. While we did not test specifically for protein aggregation in eosinophilic *Mtrr*^*gt/gt*^ female livers, an up-regulation of the *Ddit3* gene implicates ER stress. Whether this is a primary or secondary effect of the *Mtrr*^*gt*^ allele is unclear. Further experimentation is required to more fully understand how the *Mtrr*^*gt/gt*^ mutation causes eosinophilic hepatocytes and its relationship to cell stress.

Given that homocysteine aids TAG accumulation [70,71], one reason why perturbation of one‑carbon metabolism alters lipid content and hepatosteatosis is the presence of hyperhomocysteinemia. Others have shown via reduced representation bisulfite sequencing analysis that *Mtrr*^*gt/gt*^ livers have patterns of differential methylation of DNA compared to wildtype littermates [72]. Some of the affected genomic regions included genes associated with lipid metabolism [72], though the functional relevance of this differential methylation is yet-to-be determined. Despite increased plasma total homocysteine and altered liver DNA methylation in *Mtrr*^*gt/gt*^ mice, the only detectable effect of *Mtrr*^*gt/gt*^ mutation on lipid metabolism was reduced β-oxidation of fatty acids in female livers. While hepatic fatty acid oxidation was also impaired in rats fed a methyl-donor deficient diet, these rats also displayed carnitine deficit and liver steatosis [73], features that were absent in *Mtrr*^*gt/gt*^ livers. In fact, lipid content and metabolism was within the normal range in *Mtrr*^*gt/gt*^ female and male livers.

NAFLD and glycogen storage disease are often accompanied by alterations in mitochondrial OXPHOS and/or ETS function [[37], [38], [39], [40]]. Furthermore, folate metabolism is essential for the methylation of mitochondrial tRNA required for successful mRNA translation in the mitochondria [74] to ensure respiratory complexes for OXPHOS are functioning [75]. No difference in *J*O_2_ in any of the states assayed was observed in *Mtrr*^*gt/gt*^ liver. This result is consistent with only subtle changes in lipid and glycogen metabolism and demonstrates that hepatic mitochondrial function was unaltered by the *Mtrr*^*gt/gt*^ mutation.

It is unclear why folate deficiency and not the *Mtrr*^*gt/gt*^ mutation cause steatosis of the liver. The *Mtrr*^*gt*^ allele in mice is robust enough to cause many of the same phenotypes as humans with *MTRR* mutations or folate deficiency (e.g., hyperhomocysteinemia, anaemia, neural tube defects, fetal growth restriction) [21,30,[32], [33], [34], [35], [36]]. The macrocytic anaemia phenotype in *Mtrr*^*gt/gt*^ female mice is late-onset at 22 weeks of age [36]. It is possible that liver disease also appears later in life in *Mtrr*^*gt/gt*^ mice and was not apparent in our study since 9–16 week-old mice were assessed. Alternatively, the *Mtrr*^*gt*^ allele is a knockdown rather than knockout mutation, with 24% of wildtype *Mtrr* transcripts remaining in *Mtrr*^*gt/gt*^ female livers. This level of expression might be sufficient for lipid metabolism to proceed and a conditional knockout of *Mtrr* expression specific to the liver might be required to induce more severe effects on lipid metabolism and steatosis. More severe metabolic liver phenotypes may also be uncovered through exposing the *Mtrr*^*gt/gt*^ mouse to a high fat diet (e.g. [31]), a NASH-inducing diet containing trans-fats, (e.g [76]), or a folate-deficient diet (e.g. [77]).

## Appendix A Supplementary data

Supplementary material 1 Other lipid species in male and female Mtrr^gt/gt^ mouse liver. Supplementary material 1

Supplementary material 2 Triacylglycerol species in male and female C57Bl6/J control and Mtrr^gt/gt^ mouse liver. Supplementary material 2
