# Supplementary figures and images for: Mtrr hypomorphic mutation alters liver morphology, metabolism and fuel storage in mice
Source: Mol Genet Metab Rep. 2020 Mar 24;23:100580. doi: 10.1016/j.ymgmr.2020.100580 (PMC7109458; doi:10.1016/j.ymgmr.2020.100580)

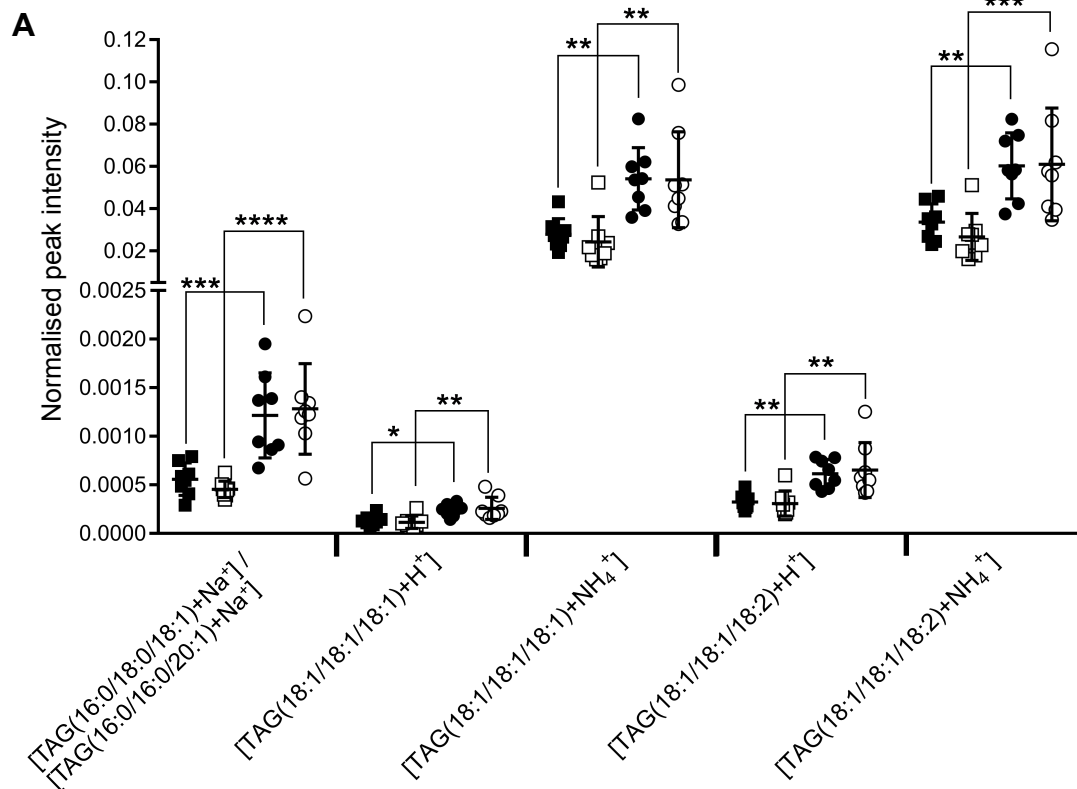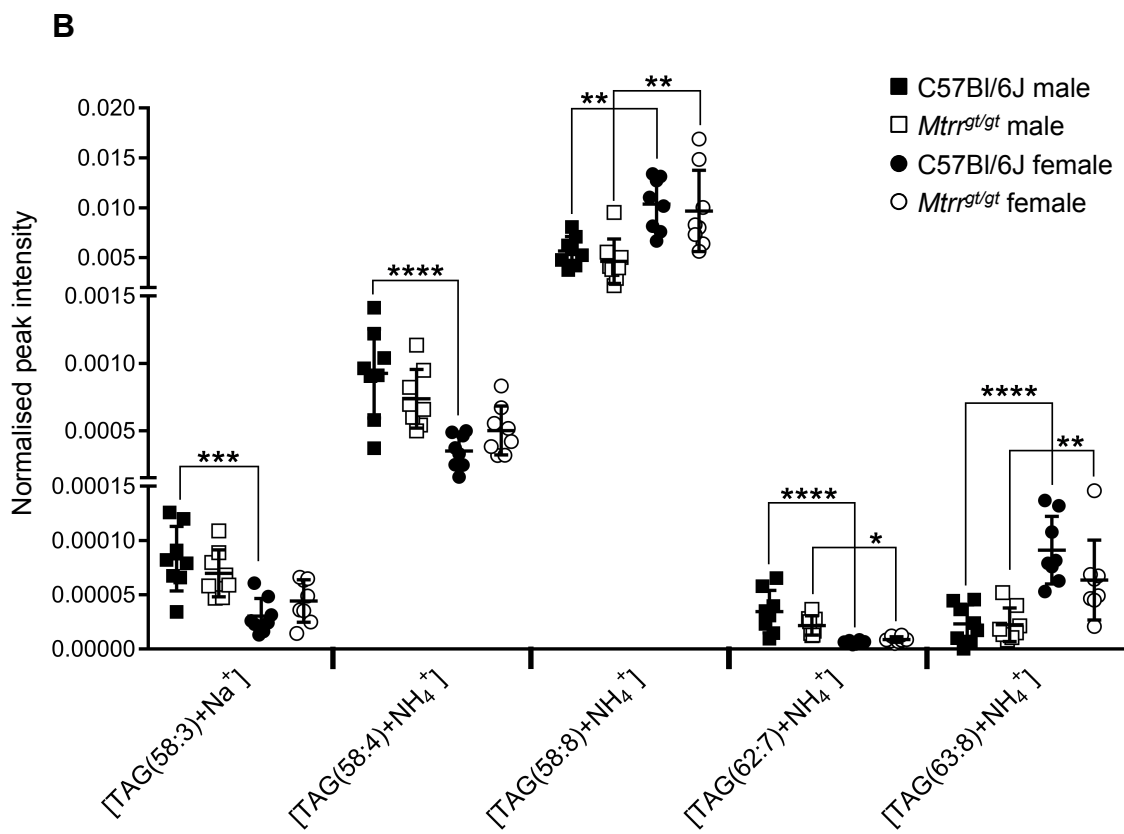

Supplement: Supplementary material 1 — Other lipid species in male and female Mtrrgt/gt mouse liver. [file mmc1.pdf]

**A**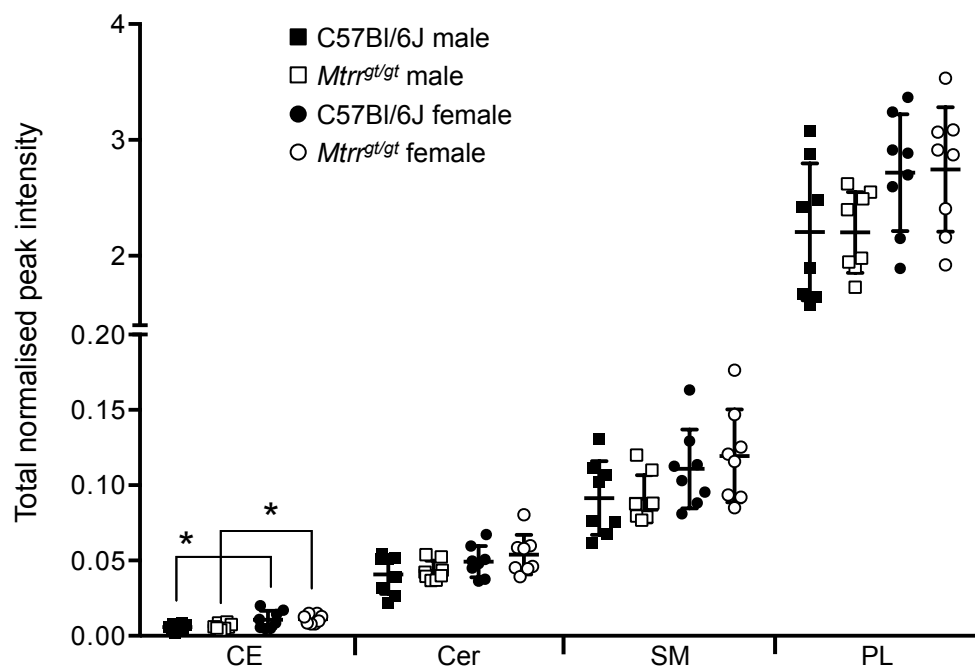**B**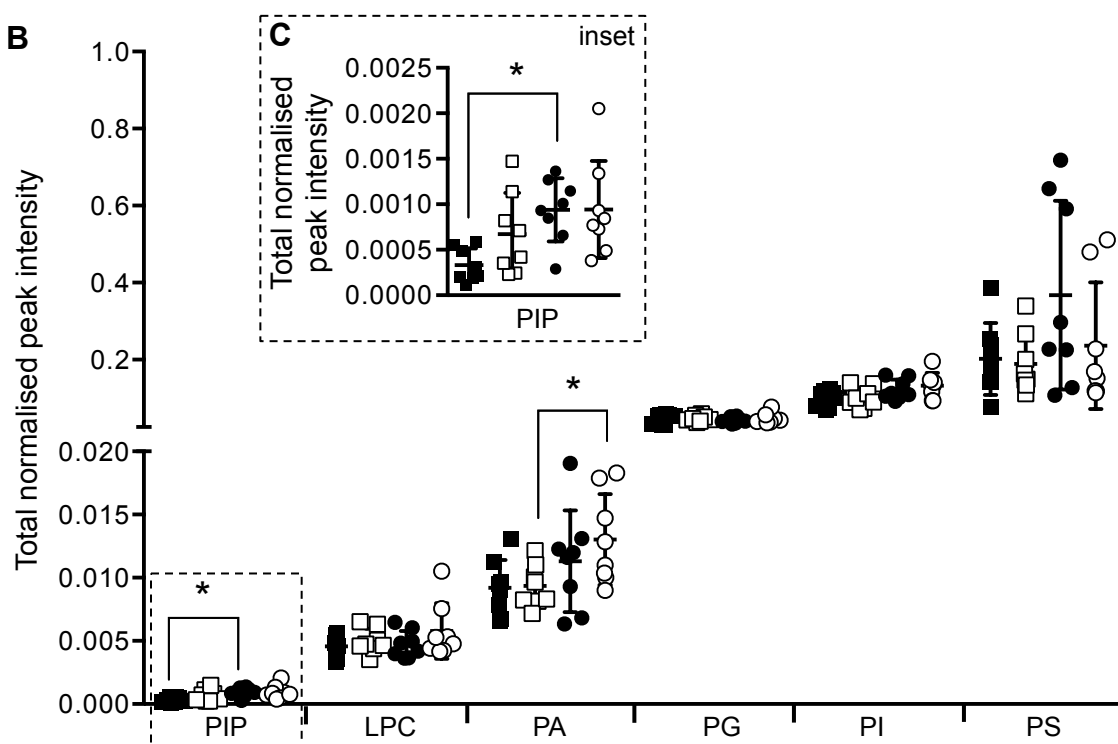

Supplement: Supplementary material 2 — Triacylglycerol species in male and female C57Bl6/J control and Mtrrgt/gt mouse liver. [file mmc2.pdf]
