# Supplementary material for: Mtrr hypomorphic mutation alters liver morphology, metabolism and fuel storage in mice
Source: Mol Genet Metab Rep. 2020 Mar 24;23:100580. doi: 10.1016/j.ymgmr.2020.100580 (PMC7109458; doi:10.1016/j.ymgmr.2020.100580)
Supplement: Supplementary material 3 [file mmc3.docx]

**Supplementary Figure Legends**

**Supplementary Fig 1. Other lipid species in male and female *Mtrr^gt/gt^* mouse liver.**

Other lipid species measured through open profiling lipidomics by liquid chromatography-mass spectrometry in male (m) and female (f) livers from C57Bl/6J control and *Mtrr^gt/gt^* mice. Data are normalised to an appropriated internal standard and sample protein concentration. (**A**) Cholesteryl-esters (CE), ceramides (Cer), sphingomyelins (SM) and phospholipids (PL). (**B**) Phospholipid species, such as phosphatidic acid (PA), lysophosphatidylcholine (LPC), phosphatidylglycerol (PG), phosphatidylinositol (PI), phosphatidylinositol phosphate(s) (PIP) and phosphatidylserine (PS). (C) PIP levels are shown as an expanded inset. N=8 livers per group, mean ± sd. *p<0.05, two-way ANOVA, Sidak’s multiple comparison. Squares, males; Circles, females; black, C57Bl/6J; white, *Mtrr^gt/gt^*.

**Supplementary Fig 2. Triacylglycerol species in male and female C57Bl/6 control and *Mtrr^gt/gt^*** **mouse liver.**

(**A-B**) Triacylglycerol (TAGs) species measured through open profiling lipidomics by liquid chromatography-mass spectrometry in male (m) and female (f) livers from C57Bl/6J control and *Mtrr^gt/gt^* mice. Data are normalised to an appropriated internal standard and sample protein concentration. Specific fatty acid composition was identified through fragmentation analysis. (**A**) Graph showing medium-chain TAGs (48-54 carbons). 16:0, palmitic acid; 18:0, stearic acid; 18:1, oleic acid; 18:2, linoleic acid; 20:1, eicosenoic acid. (**B**) Graph showing long-chain TAGs (>54 carbons). N=8 livers per group. Data is shown as mean ± sd. Two-way ANOVA with Holm-Sidak correction for false discoveries, Sidak’s multiple comparison. *p<0.05, **p<0.01, ***p<0.001, ****p<0.0001. Squares, males; Circles, females; black fill, C57Bl/6J; white fill, *Mtrr^gt/gt^*.

**Supplementary Tables**

**Supplementary table 1.** Primer sequences (mouse) used for RT-qPCR.

| **Gene** | **Forward primer (5’→3’)** | **Reverse primer (5’→3’)** | **Ref.** |
| --- | --- | --- | --- |
| *Actb* | CCCTAAGGCCAACCGTGAA | CAGCCTGGATGGCTACGTACA | [1] |
| *Agl* | ATACCTGGAGTGACGTTGGG | TGTATGCAGATTCCGGGTGT | - |
| *Bhmt* | ACATCAGGGCGATTGCAGA | CGGGACATGGAAGGGTTG | [2] |
| *Bhmt2* | CGGATTTGAGCCCTACCACA | AGCAGATTCTCCCAGTATTCT | [2] |
| *Cebpa* | TGGACAAGAACAGCAACGAG | GTCACTGGTCAACTCCAGCA | [3] |
| *Ddit3* | GGAGGTCCTGTCCTCAGATG | GGACGCAGGGTCAAGAGTAG | - |
| *G6pc* | ATCAATCTCCTCTGGGTGGC | GCTGTAGTAGTCGGTGTCCA | - |
| *Gbe1* | CCGAGGGACCCATGATCTTT | ACACCATCAAAACGGAAGCC | - |
| *Gsk3a* | AGCCCCAGAATTTGCTTGTG | GAGTTCTGGAGCACGGTAGT | - |
| *Gsk3b* | AGCCACTGATTACACGTCCA | ACCAACTGATCCACACCACT | - |
| *Gyg* | ACCCTCCATTGAAACGTATAACC | CGTTGCCCAGCCACTAAAAT | - |
| *Gys2* | CCTGTGTGGGAAGCTGAAAG | TTTGGCCTTGGTCTGGATCA | - |
| *Hprt* | CAGGCCAGACTTTGTTGGAT | TTGCGCTCATCTTAGGCTTT | [4] |
| *Isca1* | GAAGTTGAGTCCCCACGCTC | TTACAGCAGAGGGGGTCAGT | - |
| *Mthfr* | AGCTTGAAGCCACCTGGACTGTAT | AGACTAGCGTTGCTGGGTTTCAGA | [5] |
| *Mtr* | GCAGATGTGGCCAGAAAG | GCCACAAACCTCTTGACTCC | [5] |
| *Mtrr* (total) | GGTTTTCCGCAGATCTTCAC | CTGTGTCAGGTGGGTCTCCT | [4] |
| *Mtrr* (wildtype) | GGGAAATTTGGAGCTATGTGG | CAGATGAGTCAAGACCCCAGT | [4] |
| *Myc* | CTGCTGTCCTCCGAGTCCT | GGTTTGCCTCTTCTCCACAG | - |
| *Ndufs4* | GTACTCGCATCCTGGCGTTT | GGATGTGCTCAACAACCTGGA | - |
| *Ndufs5* | CGGGCTTGCTGAAAAAGACA | TGGCAAAGTTCTCATTCACCTCT | - |
| *Ndufs6* | GGGTTTCGGGGTTCAAGTGT | TGGCAAAGTTCTCATTCACCTCT | - |
| *Ugp2* | TTACCACAGCAGCCTCACAT | TGGATTTTACCCCAGTCCACA | - |

**References:**

[1] Zhu J., Mackem S., Analysis of mutants with altered SHH activity and posterior digit loss supports a biphasic model for SHH function as a morphogen and mitogen Dev Dyn 240 (2011) 1303-1310.

[2] Jacobs R.L., Stead L.M., Devlin C., Tabas I., Brosnan M.E., Brosnan J.T., Vance D.E., Physiological regulation of phospholipid methylation alters plasma homocysteine in mice J. Biol. Chem. 280 (2005) 28299-28305.

[3] Kode A., Mosialou I., Silva S.C., Joshi S., Ferron M., Rached M.T., Kousteni S., FoxO1 protein cooperates with ATF4 protein in osteoblasts to control glucose homeostasis J. Biol. Chem. 287 (2012) 8757-8768.

[4] Padmanabhan N., Jia D., Geary-Joo C., Wu X., Ferguson-Smith A.C., Fung E., Bieda M.C., Snyder F.F., Gravel R.A., Cross J.C., Watson E.D., Mutation in folate metabolism causes epigenetic instability and transgenerational effects on development Cell 155 (2013) 81-93.

[5] Uthus E.O., Brown-Borg H.M., Methionine flux to transsulfuration is enhanced in the long living Ames dwarf mouse Mech Ageing Dev 127 (2006) 444-450.

**Supplementary table 2.** Internal standards used for liquid chromatography-mass spectrometry analysis of lipid and acyl-carnitine species.

| **Internal Standard** | **Concentration** | **Assay** | **Vendor** |
| --- | --- | --- | --- |
| C16-d31 ceramide | 2.5 μgml^-1^ | Lipidomic open profiling | Avanti Polar Lipids, Inc. |
| 16:0-d31-18:1 phosphatidic acid |  |  |  |
| 16:0-d31-18:1 phosphatidylcholine |  |  |  |
| 16:0-d31-18:1 phosphatidylethanolamine |  |  |  |
| 16:0-d31-18:1 phosphatidylglycerol |  |  |  |
| 16:0-d31-18:1 phosphatidylinositol |  |  |  |
| 14:0 phosphatidylserine d54 |  |  |  |
| 16:0-d31 sphingomyelin |  |  |  |
| 18:0-d6 cholesteryl ester | 2.5 μgml^-1^ | Lipidomic open profiling | CDN Isotopes |
| 15:0-d29 fatty acid |  |  |  |
| 17:0-d33 fatty acid |  |  |  |
| 20:0-d39 fatty acid |  |  |  |
| 14:0-d29 lysophosphatidylcholine-d13 |  |  |  |
| 45:0-d87 triglyceride |  |  |  |
| 48:0-d83 triglyceride |  |  |  |
| 54:0-d105 triglyceride |  |  |  |
| Free carnitine-d9 | 2 μM | Carnitine assay | Cambridge Isotope Laboratories, Inc. |
| Acetylcarnitine-d3 |  |  |  |
| Propyonilcarnitine-d3 |  |  |  |
| Butyrylcarnitine-d3 |  |  |  |
| Isovalerylcarnitine-d9 |  |  |  |
| Octanoylcarnitine-d3 |  |  |  |
| Myristoylcarnitine-d9 |  |  |  |
| Palmitoylcarnitine-d3 |  |  |  |
